# Ultra-high pressure processing enhances structural, rheological and *in vitro* digestibility properties of rice starch‑calcium gluconate complexes

**DOI:** 10.1016/j.fochx.2025.103220

**Published:** 2025-10-28

**Authors:** Sixuan Zhao, Xinhua He, Yue Wang

**Affiliations:** a College of Food Science and Engineering, Gansu Agricultural University, Lanzhou 730070, China

**Keywords:** Starch‑calcium gluconate complexes, Ultra-high pressure (UHP), Structural characteristics, Rheological properties, *In vitro* digestibility

## Abstract

Calcium gluconate (CG) is widely used as both a nutrient fortifier and starch modifier. This study pioneers the application of ultra-high pressure (UHP) processing for the preparation of rice starch-CG complexes, in comparison to conventional hydrothermal (H) treatment. The structural characteristics, rheological properties and *in vitro* digestibility of the complexes were systematically investigated. UHP at 500 MPa yielded complexes with markedly lower pasting viscosity but superior gel fluidity and syneresis stability, ideal for smooth semi-solid foods requiring controlled water release. Meanwhile, UHP at 200 MPa increased resistant starch (RS) content to 36.00 %, significantly surpassing H results and highlighting its potential in low-digestibility functional foods. Although both methods improved solubility and reduced viscoelasticity, UHP enabled precise, pressure-dependent modulation of starch functionality, which is not attainable through conventional heating. These findings demonstrate UHP's potential for tailoring starch functionality, offering new strategies for developing semi-solid foods and low-digestibility foods through precise pressure selection.

## Highlights

• Pioneering use of ultra-high pressure (UHP) for starch-calcium gluconate complexes.
• Calcium gluconate boost solubility, gel fluidity, and resistant starch (RS) content.
• 500 MPa UHP yields lowest viscosity, best fluidity; 200 MPa maximizes RS (36 %).
• UHP-prepared Ca^2+^-starch enables semi-solid & low-digestibility foods applications.

## 1 Introduction

Rice starch, a major component of rice, is widely used in the food industry, especially as a thickener, stabilizer, gelling agent and water-holding ingredient, to enhance the viscosity, physicochemical properties and overall stability of food products. Therefore, starch is often regarded as a key filler in food processing, contributing to improved product quality and gel strength regulation (Chu et al., 2019). However, native rice starch exhibits several limitations, such as poor freeze-thaw stability, a tendency to paste easily, low solubility in cold water, inadequate heat and shear resistance as well as a low content of resistant starch (RS), with these different drawbacks restricting its broader application in the food industry (Kim et al., 2017). To overcome these limitations, various starch modification techniques are usually employed to meet the specific requirements of food processing.

Calcium salts, widely recognized as important nutritional supplements, are commonly used to fortify starchy food products, such as hanging noodles and bread, enhancing their nutritional value. Furthermore, the incorporation of calcium salts during starch processing has been shown to improve key functional properties of starch, including viscoelasticity, digestibility, rheological behavior and thermal stability. Consequently, calcium ions are frequently utilized in starch modification research (Rossi et al., 2020; Samutsri & Suphantharika, 2012). For instance, Min et al. (2023) reported that the addition of calcium salts reduced the viscoelastic modulus and gel strength of mung bean starch. Similarly, through simulated *in vitro* digestion experiments, Jiang et al. (2024) demonstrated that hydrogel beads, formed from calcium salt-cross-linked starch, could limit enzyme-substrate interactions, thereby reducing starch digestibility, with this feature being potentially valuable for developing food products suitable for diabetic individuals. Among various calcium supplements, calcium gluconate (CG), an organic calcium salt, stands out for its promising potential in food fortification, particularly for its low cost, good water solubility and high bioavailability (Codină et al., 2018).

In the field of food processing, novel non-thermal technologies have been continuously emerging, and among these, ultra-high pressure (UHP) treatment has garnered significant attention due to its unique advantages. Indeed, compared with traditional hydrothermal treatments, UHP-based ones offer shorter processing times, lower energy consumption and superior preservation of flavor and nutrients (Pei et al., 2023; Zhang et al., 2024). Additionally, other commonly used methods for preparing starch‑calcium composites, such as ultrasonic treatment, chemical cross-linking, and enzymatic modification, also have certain limitations. Ultrasonic modification typically requires extremely precise process control, involves uncontrollable factors, and often necessitates combination with other techniques to produce effective composites (Wang et al., 2024); Chemical cross-linking methods frequently present issues such as low safety, complex processes, high costs, and potential food safety concerns (Liu, Li, et al., 2018); Enzymatic modification is constrained by factors including the inherent instability of enzymes, susceptibility to environmental interference, low reaction efficiency, prolonged reaction times, and stringent starch pretreatment requirements, making large-scale production challenging (Zhu et al., 2019). In contrast, UHP technology eliminates the need for harmful chemicals, modifying starch through purely physical means with precise pressure regulation, thereby providing a more environmentally sustainable approach (Carpentieri et al., 2024; Castro et al., 2020). Research has shown that starch can undergo gelatinization under high-pressure conditions, and even though high-pressure-induced starch pasting shares several similarities with the heat-induced process, significant differences can also be observed. Notably, UHP-treated starch can exhibit a lower retrogradation rate, a more fragmented granule structure as well as reduced viscosity in its aqueous dispersions. These particular characteristics suggest that UHP-treated starch may contribute positively to the textural attributes of food products during processing and storage (Liu et al., 2020; Mirzababaee et al., 2022). In this context, Carpentieri et al. (2024) reported that UHP-treated rice and tapioca starches could exhibit enhanced flow properties and digestibility, making them suitable for use in cosmeceutical, pharmaceutical and food applications. Additionally, Thakur et al. (2024) demonstrated that UHP treatment could improve the functional and gelling properties of red quinoa, enabling its use as a gelling or curing agent in food systems. However, despite growing interest in UHP as a starch modification technique, limited research has focused on the preparation of starch‑calcium ion complexes using this method.

As one of the most mature and widely used starch modification methods, hydrothermal (H) treatment provides a foundational reference. H treatment primarily relies on thermal energy as its driving force, which results in a relatively simple process that struggles to achieve precise functional differentiation. In contrast, UHP treatment enables distinct functional outcomes through the precise selection of pressure parameters. Furthermore, UHP can modify starch at room temperature, helping to preserve heat-sensitive components in food. This opens up new possibilities for developing novel functional foods. Rios-Corripio et al. (2020) found that UHP treatment significantly enhanced the antioxidant activity, total phenolic compounds, flavonoids, and anthocyanin content of fermented pomegranate beverages. Ding, Ban, et al. (2023) indicated that UHP processing significantly enhanced the nutritional value of grains and legumes (such as the bioavailability of vitamins and minerals) and bio-functional properties (*e.g.*, production of bioactive peptides, increasing the content of γ-aminobutyric acid). In their review, Tewari et al. (2017) noted that fruits, vegetables, and their products subjected to high-pressure processing exhibit superior antioxidant activity compared to thermally processed foods. A systematic comparison between the emerging non-thermal UHP technology and this classical thermal approach allows for a comprehensive evaluation of UHP's potential advantages and unique effects on the formation of starch‑calcium complexes and the regulation of their functional properties. Most current research focuses on H treatment for preparing starch-mineral composites, while studies utilizing non-thermal UHP processing technology to synergistically regulate starch‑calcium ion complexation remain unexplored. UHP not only induces starch gelatinization, but its hydrostatic pressure characteristics may also influence the microstructure, physicochemical properties, and digestibility of starch molecules in ways distinct from heat methods. Hence, it is essential to study the physicochemical, rheological and digestive properties of rice starch‑calcium salt complexes produced through UHP processing. To this end, this study aimed to investigate the microstructure, physicochemical characteristics and digestibility of such complexes before comparing them with those prepared by conventional hydrothermal treatment. It is expected that the findings will provide a theoretical basis for expanding the application of rice starch in food processing.

## 2 Materials and methods

### 2.1 Materials

Native rice starch (NS), with a water content of 9.28 % and an amylose content of 22.31 %, was obtained from Wuxi Jinnong Technology Co. Ltd. (Wuxi, China), while calcium gluconate (CG, purity ≥99.00 %) was purchased from Yuan Ye Biotechnology Co. (Shanghai, China). All other reagents used in this study were of analytical grade.

### 2.2 Preparation of starch‑calcium gluconate complexes under UHP treatment

Rice starch (10.0 g) and CG (added at 5.40 % of the starch weight, w/w) were dispersed uniformly in 100 mL of deionized water (Lu et al., 2023). The resulting suspension was then vacuum-sealed in aluminum foil pouches before being subjected to UHP treatments at 200, 400 and 500 MPa for 10 min at 25 °C (Zhang et al., 2023) (Huataisenmiao High-Pressure Technology Co., Ltd., China), with these samples eventually designated as 200 MPa-CG (Partial starch gelatinization), 400 MPa-CG (starch incompletely gelatinized) and 500 MPa-CG (starch completely gelatinized), respectively. Parallel control groups without CG were also included and labeled as 200 MPa-Control, 400 MPa-Control and 500 MPa-Control. The pretreatment procedures for starch suspensions were identical for both H and UHP treatments. Specifically, 10.0 g of rice starch and CG (based on 5.4 % starch, w/w) were uniformly dispersed in 100 mL of deionized water. After being thoroughly mixed with a glass rod, the sample was incubated in an 85 °C water bath for 10 min and labeled as H-Control and H-CG. All treated starches were dried at 45 °C for 24 h before subsequent analyses.

### 2.3 Scanning electron microscopy (SEM)

The starch samples were mounted onto SEM stubs using double-sided adhesive tape, and following gold-sputter coating, the surface morphology of the granules was observed at a magnification of 5000× with a scanning electron microscope (JSM-6701F; Electron Optics Inc., Japan) (Sherin et al., 2024).

### 2.4 Particle size analysis

Particle size distribution was determined using a laser diffraction particle size analyzer (Bettersize 2600 System, Dandong Baite, China). For this purpose, the starch granules were first uniformly dispersed in deionized water before being subjected to ultrasonic agitation. To ensure accurate measurements, the shading rate was adjusted to fall within the range of 0.80 % to 1.20 % (Liu, Wu, et al., 2018).

### 2.5 Differential scanning calorimetry (DSC)

Each starch sample (2.5 mg) was weighed into an aluminum crucible, after which deionized water was added in a ratio of 1:3. The crucible was then sealed and equilibrated at room temperature for 24 h prior to thermal analysis using a differential scanning calorimeter (Q20; TA Instruments, New Castle, DE, USA). The test was performed from 30 °C to 120 °C at a heating rate of 10 °C/min (Guo et al., 2015).

### 2.6 X-ray diffraction (XRD) analysis

The crystalline structure of rice starch was analyzed using XRD (XRD-6000, Shimadzu, Japan) according to the method of Zhou et al. (2022). Scans were conducted over a 2θ range of 5° to 40°, with a step size of 0.02°.

### 2.7 Water and oil absorption capacity (WAC and OAC)

Starch samples (1.0 g) were thoroughly mixed with 10 mL of either deionized water or purified soybean oil. The resulting mixtures were then stirred for 1 h at room temperature, and after a 10-min centrifugation at 3000 rpm, the WAC and OAC were calculated using the following equations:

(1) $$\mathrm{WAC}\;\mathrm{or}\;\mathrm{OAC}\;\left(g/g\right)=M_{2}/M_{1}$$

where, M_1_ is the weight of the dry starch sample and M_2_ is the weight of the wet sediment after centrifugation (Mirzababaee et al., 2022).

### 2.8 Swelling power and solubility

Swelling power and solubility were measured according to the method of Li et al. (2018), with slight modifications. Briefly, each starch sample (0.5 g) was dispersed in 25 mL of distilled water in a centrifuge tube before being heated in a water bath at 70 °C for 30 min. After cooling to room temperature, the resulting starch paste was centrifuged at 3500 rpm for 30 min, with the supernatant subsequently collected and dried to a constant weight (W_s_) in an oven at 105 °C. The precipitate was also weighed (W_p_) and dried at 105 °C to a constant weight (W_d_). The swelling power and solubility were eventually calculated as follows.

(2) $$\mathrm{Swelling Power}=W_{p}/W_{d}$$

(3) Solubility=Ws/0.5×100

### 2.9 Rheological properties

Rheological measurements were performed using a DHR rheometer (DHR-1 rheometer, TA instrument, DE, USA). Each starch sample (0.5 g) was mixed with 6 mL of deionized water and heated in a boiling water bath for 30 min to form a gel. The latter was then cooled and loaded onto the rheometer using a 40 mm diameter parallel plate with a 1.0 mm gap. Dynamic frequency sweeps were conducted within the linear viscoelastic at 2 % strain across a frequency range of 0.1 to 100 rad/s (Kaur et al., 2019).

### 2.10 Gel strength measurements

The gel texture properties of starch were determined using the method of Hu et al. (2020). Samples were prepared as 15 % (w/w) starch suspensions and heated in a boiling water bath for 20 min. After gelatinization, the samples were removed, cooled to room temperature, stored overnight at 4 °C, and then used for texture analysis. Gel strength was measured using a cylindrical probe (P/0.5) on a TA-XT Plus texture analyzer (Stable Micro Systems, Robbinsville, NJ). Test parameters were: probe descent speed 1.0 mm/s, test speed 1.0 mm/s, probe ascent speed 1.0 mm/s, test height 4 mm, and trigger force 4.0 g.

### 2.11 Steady shear viscosity measurements

Steady shear viscosity was also assessed using the DHR-1 rheometer (TA instrument, DE, USA). In this case, the starch gels were placed on the measurement platform with a plate diameter of 60.0 mm and a gap of 1.0 mm. Measurements were then conducted at 25 °C, with a shear rate sweep ranging from 0.1 to 100 s^−1^ (Qian et al., 2019).

### 2.12 Freeze-thaw stability

The freeze-thaw stability of the starch samples was assessed based on the method of Li et al. (2024), with slight modifications. For each sample, a 5 % (*w*/w, dry basis) starch suspension was prepared and heated in a boiling water bath for 30 min. The resulting gel was then frozen at −20 °C for 24 h before being thawed at 30 °C for 2 h. After thawing, the samples were centrifuged at 3000 rpm for 10 min, and the amount of separated water was used to calculate the dehydration shrinkage. The freeze-thaw stability was determined over four complete freeze-thaw cycles.

### 2.13 *In vitro* digestibility of starch samples

*In vitro* digestibility of the starch samples was assessed based on the method of Chen et al. (2024), with slight modifications. Each starch sample (100 mg) was dispersed in 5 mL of simulated saliva and incubated in a water bath at 37 °C for 10 min. This was followed by the addition of 5 mL of simulated gastric fluid (containing 3.50 mg.mL^−1^ of pepsin and 0.03 mol.L^−1^ CaCl_2_), with the pH subsequently adjusted to 3.0 using 1 M HCl prior to a 2-h incubation in a 37 °C water bath. After the gastric digestion, 5 mL of simulated intestinal fluid (containing α-amylase at 200 U.mL^−1^, glucoamylase at 160 U.mL^−1^ and 0.03 mol.L^−1^ CaCl_2_) was added, and the pH was adjusted to 7.0 using 1 M NaOH before incubating for an additional 2 h in a water bath at 37 °C. Samples (0.5 mL) were then collected after oral and gastric digestion as well as at 0, 10, 20, 30, 60, 90 and 120 min during intestinal digestion. Each collected sample was mixed with 2 mL of anhydrous ethanol to inactivate enzyme activity, and after a 10-min centrifugation at 5000 rpm, the glucose content in the supernatant was determined using the 3,5-dinitrosalicylic acid method. A standard curve was established using glucose solutions with concentrations ranging from 0.1 to 0.5 mg.mL^−1^. Absorbance was measured at a wavelength of 540 nm, yielding the standard curve equation: y = 1.1786x−0.0101. R^2^ = 0.9942. The reducing sugar content of all samples was calculated using this standard curve.

The hydrolysis index (HI), rapidly digestible starch (RDS), slowly digestible starch (SDS) and RS were calculated as follows:

(4) $$\mathrm{HI}\;\left(\%\right)=\frac{R_{t}}{W}\times 100$$

(5) $$\mathrm{SDS}\left(\%\right)=\frac{\left(R_{120}-R_{20}\right)}{W}\times 0.9\times 100$$

(6) $$\mathrm{RDS}\left(\%\right)=\frac{\left(R_{20}-D\right)}{W}\times 0.9\times 100$$

(7) $$\mathrm{RDS}\left(\%\right)=100-\mathrm{RDS}-\mathrm{SDS}$$

where R_t_ is the amount of reducing sugar (mg glucose equivalents**)** at time t, R_120_ and R_20_ are the reducing sugar contents (mg glucose equivalents) at 120 min and 20 min of intestinal digestion, respectively, D is the amount of free reducing sugar (mg glucose equivalents) at the beginning of digestion, and W is the total dry mass of starch (mg). The factor 0.9 accounts for the conversion from glucose to starch equivalents.

### 2.14 Determination of calcium content

Following *in vitro* digestion, the calcium content was determined using atomic absorption spectroscopy (novAA400P, Analytik Jena AG) as described by Wu et al. (2023), with slight modification. A 5-mL aliquot of the post-digestion supernatant was mixed with 5 mL of HNO_3_ as well as 2 mL of H_2_O_2_, and after complete digestion, the sample was transferred to a 25-mL volumetric flask and diluted to the mark prior to measurement.

### 2.15 Statistical analysis

All experiments were performed in triplicate and statistically analyzed with SPSS 26.0 software. Differences between means were assessed using Duncan's multiple range test (*p* < 0.05), with graphs subsequently generated using origin 2019 software.

## 3 Results and discussion

### 3.1 SEM analysis

**Fig. 1:**
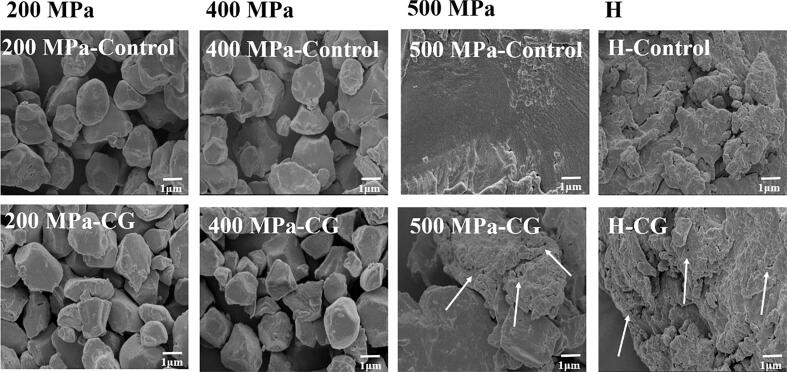
SEM images of native rice starch and starch-CG complexes under different treatment conditions.

The SEM images of all samples are presented in Fig. 1. Native rice starch granules were found to be small, with a polygonal polyhedral shape and a smooth surface. Furthermore, under UHP treatment at 200 MPa and 400 MPa, no significant morphological changes were observed, indicating that the starch granule structure remained largely intact or was only minimally disrupted under low pressure conditions. However, when the pressure was increased to 500 MPa, the starch granules exhibited severe structural damage and appeared gelatinized. These findings were consistent with those of Sherin et al. (2024) who reported complete gelatinization of millet starch at 600 MPa. Similarly, the conventional H treatment completely destroyed the granule structure, resulting in a gelatinous appearance. This was comparable to the morphological changes observed under 500 MPa pressure. This observation aligned with that of Oh et al. (2019) who also noted that H treatment could disrupt the structure of starch granules. In addition, numerous small pore-like structures appeared on the surfaces of CG-treated starch granules under both 500 MPa and H conditions. This effect was likely due to high pressure or temperature, which led to starch gelatinization and a looser surface structure. At the same time, by altering surface charge distribution, Ca^2+^ ions may interfere with hydration and induce localized dehydration and contraction, ultimately promoting pore formation (Fei et al., 2025).

### 3.2 Particle size distribution

The particle size distribution of the starch samples is summarized in Table 1. Overall, compared with their respective controls, the addition of CG reduced the particle size of rice starch in both UHP-treated and H-treated groups. This effect could have been due to electrostatic interactions whereby the positively charged Ca^2+^ ions neutralized the negatively charged rice starch molecules, thereby reducing inter-molecular repulsion. Furthermore, Ca^2+^ ions may induce unfolding of the starch molecules, contributing to a decrease in their granule size (Liu et al., 2024). The particle size was observed to decrease in the order of 500 MPa > H > 400 MPa > 200 MPa. The treatments at 200 MPa and 400 MPa did not significantly alter particle size distribution. In contrast, starch granules treated at 500 MPa exhibited a significant increase in particle size (*P* < 0.05), likely due to granule gelatinization which caused swelling and aggregation (Zhang, He, et al., 2021). The results from the H treatment were also consistent with those of the 500 MPa group, thus further supporting the conclusion that both high-pressure and high-temperature conditions promoted complete starch gelatinization, as previously reported by Oh et al. (2019). In addition, under high-pressure (500 MPa) or high-temperature processing conditions, starch undergoes complete gelatinization, during which the granular structure disintegrates and releases starch molecular chains. The liberated Ca^2+^ ions serve as cross-linking bridges, promoting the recombination and polymerization of the released amylose and amylopectin chains, ultimately leading to a reduction in particle size. (Min et al., 2023).

**Table 1:** Effects of CG on the particle size distribution of rice starch under different treatment conditions.

Samples	D[4,3]/(μm)	D10/(μm)	D50/(μm)	D90/(μm)
200 MPa-Control	5.75 ± 0.17^e^	2.01 ± 0.02^c^	5.46 ± 0.09^e^	9.29 ± 0.31^f^
200 MPa-CG	5.61 ± 0.24^e^	2.04 ± 0.04^c^	5.53 ± 0.17^e^	9.03 ± 0.51^f^
400 MPa-Control	7.32 ± 0.50^e^	2.28 ± 0.08^c^	6.26 ± 0.20^e^	13.62 ± 1.54^e^
400 MPa-CG	5.98 ± 0.27^e^	2.06 ± 0.02^c^	5.41 ± 0.08^e^	9.61 ± 0.36^f^
500 MPa-Control	131.90 ± 2.16^a^	10.07 ± 0.55^a^	135.97 ± 2.49^a^	249.00 ± 1.84^b^
500 MPa-CG	89.40 ± 1.65^c^	7.40 ± 0.76^b^	73.10 ± 3.19^c^	204.90 ± 2.85^c^
H-Control	125.00 ± 1.61^b^	7.13 ± 0.94^b^	92.24 ± 2.06^b^	301.83 ± 3.69^a^
H-CG	71.82 ± 1.78^d^	6.62 ± 0.63^b^	32.72 ± 1.95^d^	198.97 ± 1.53^d^

Granular parameter: D [4,3], volume-weighted mean diameter; 200 MPa, 400 MPa, 500 MPa, high-pressure-treated rice starch; H, hydrothermal treatments.

Values are means ± SD (triplicate experiments); values in the same column with different superscripts denote significant differences (P < 0.05).

### 3.3 DSC analysis of thermal properties

The DSC parameters of all starch samples are presented in Table 2. Under UHP treatments at 200 MPa and 400 MPa, the addition of CG increased both the To and ΔH of rice starch, with the sample treated with CG at 200 MPa exhibiting the highest values (62.45 °C for To and 3.03 J/g for ΔH). This enhancement could be attributed to dipole-metal interactions between the hydroxyl groups of starch and calcium ions, which enhanced the molecular order within the starch granules, thereby raising the thermal stability (Kraithong et al., 2023). Furthermore, calcium ions possess a small ionic radius as well as a high charge density, which reduce the amount of free water available for starch gelatinization, resulting in a higher pasting temperature (Abedi & Pourmohammadi, 2020). In this context, Pelpolage et al. (2018) also reported that the addition of calcium ions could increase the To and ΔH of potato starch. However, as the treatment pressure increased from 200 MPa to 400 MPa, both To and ΔH decreased in the order 200 MPa > 400 MPa, with this tend suggesting that UHP treatment disrupted the intra- and intermolecular hydrogen bonding within starch molecules, making the double-helical crystalline regions more susceptible to thermal disruption (Liu, Wu, et al., 2018). Moreover, no thermal transitions (To, Tp, Tc or ΔH) were detected in samples treated at 500 MPa or subjected to H treatment. This indicated that the crystalline structure of starch was completely disrupted under these conditions, suggesting full gelatinization of the starch granules (Trinh, 2015; Zhang, Xu, et al., 2021).

**Table 2:** DSC parameters of native rice starch and starch-CG complexes under different treatment conditions.

Samples	T_O_ (°C)	T_P_ (°C)	T_C_ (°C)	ΔH (J/g)
200 MPa–Control	61.33 ± 0.07^b^	65.56 ± 0.21^b^	71.58 ± 0.16^a^	2.96 ± 0.08^ab^
200 MPa–CG	62.45 ± 0.17^a^	66.71 ± 0.05^a^	72.39 ± 0.38^a^	3.03 ± 0.06^a^
400 MPa–Control	60.37 ± 0.33^c^	64.82 ± 0.20^c^	71.29 ± 1.03^a^	2.72 ± 0.10^c^
400 MPa–CG	61.18 ± 0.88^bc^	66.05 ± 0.60^b^	71.99 ± 0.38^a^	2.88 ± 0.05^b^
500 MPa–Control	ND	ND	ND	ND
500 MPa–CG	ND	ND	ND	ND
H-Control	ND	ND	ND	ND
H-CG	ND	ND	ND	ND

*To,* Onset temperature; *Tp,* peak temperature; *Tc,* end temperature; *ΔH,* enthalpy.

200 MPa,400 MPa, 500 MPa, high-pressure-treated rice starch; H, hydrothermal treatments ND: not detectable.

Values are means ± SD (triplicate experiments); values in the same column with different superscripts denote significant differences (*P* < 0.05).

### 3.4 X-ray diffraction

**Fig. 2:**
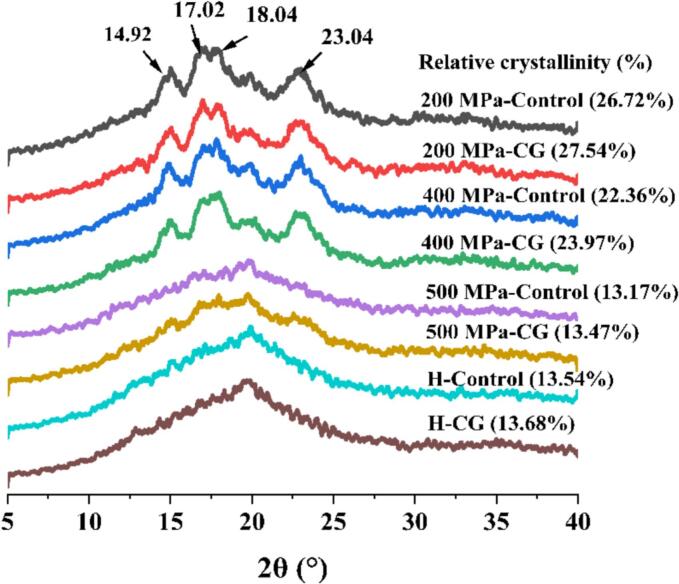
XRD patterns of native rice starch and starch-CG complexes. The values in parentheses indicate relative crystallinity (RC, %).

The XRD patterns of rice starch-CG complexes subjected to UHP and H treatments are presented in Fig. 2. Overall, native rice starch exhibited distinct diffraction peaks at 2θ angles of 15°, 17°, 18° and 23°, which were characteristic of an A-type crystalline structure. Furthermore, under 200 MPa and 400 MPa treatments, the addition of calcium salts did not alter the crystalline structure pattern of starch, indicating that the crystal type remained unchanged. However, the relative crystallinity (RC) of starch increased with the addition of CG, reaching a maximum of 27.54 % at 200 MPa. This enhanced crystallinity was likely due to cross-linking interactions between Ca^2+^ ions and starch molecules, which resulted in a more ordered molecular arrangement (Min et al., 2023). When the pressure increased to 500 MPa, all characteristic diffraction peaks disappeared, and the RC value dropped sharply from 27.54 % to 13.17 %, indicating complete disruption of the crystalline structure (Mirzababaee et al., 2022). A similar pattern was observed under H treatment, suggesting that both high pressure and high temperature can significantly damage the internal crystalline structure of rice starch (Zhang, Xu, et al., 2021). These results were consistent with the DSC data.

### 3.5 Oil and water absorption capacities

**Fig. 3:**
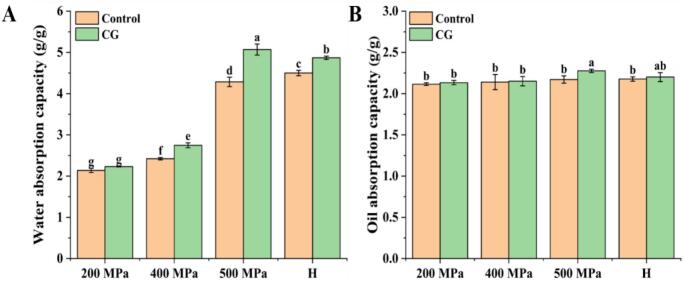
Water absorption (A) and oil absorption (B) characteristics of native rice starch and starch-CG complexes.

The OAC and WAC of all samples are shown in Fig. 3. Overall, the addition of CG significantly increased the WAC of native rice starch under both UHP and H treatments, while the OAC remained relatively unchanged. The improved water absorption could be attributed to the strong interaction between Ca^2+^ ions and the ring oxygen atoms in the anhydroglucose units of starch. These interactions disrupt intermolecular hydrogen bonding, thereby exposing more hydrophilic sites and increasing the starch's affinity for water (Abedi & Pourmohammadi, 2020). For the UHP-treated samples, the water absorption increased progressively with pressure, ranging from 2.14 g/g (200 MPa-Control) to 5.07 g/g (500 MPa-CG). The significant increase at 500 MPa could be attributed to the pressure-induced destruction of the crystalline regions, which facilitated water penetration (Sherin et al., 2024). The H-treated group also exhibited similar trends, possibly due to thermal disruption of hydrogen bonding between the crystalline and amorphous regions of starch, resulting in enhanced water uptake (Sindhu et al., 2019).

### 3.6 Swelling power and solubility

**Fig. 4:**
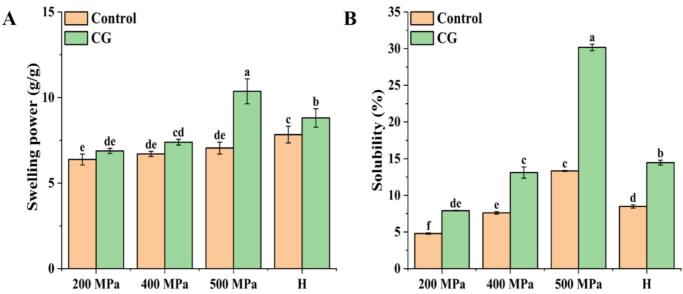
Swelling power (A) and solubility (B) of native rice starch and starch-CG complexes under different treatment conditions.

The solubility and swelling power of rice starch-CG complexes are shown in Fig. 4. In all treatments (UHP and H), the addition of CG led to significant increases in both properties compared with the control. Specifically, the 500 MPa-CG sample exhibited the highest values, with a solubility of 30.17 % and a swelling power of 8.80 g/g. This observation could be attributed to the hydrophilic nature of CG which can form hydrated ions that adsorb to the surface of starch granules, forming a hydration layer. At the same time, smaller starch particles result in thicker hydration layers, thus enhancing both solubility and swelling capacity (Abedi & Pourmohammadi, 2020). For the UHP and H treatments, swelling power and solubility increased with treatment pressure in the order 200 MPa < 400 MPa < H < 500 MPa. The high-pressure treatment group (500 MPa) exhibited significantly higher solubility and swelling than both the low-pressure treatment groups (200 MPa, 400 MPa) and the H treatment group. This trend reflected the increasing structural damage to starch granules under high pressure, resulting greater molecular fragmentation and an increase in the proportion of water-soluble starch components (Li et al., 2018). Similar results were observed under H treatment where high temperature disrupted the hydrogen bonding between linear and branched starch chains, thus exposing hydroxyl groups that enhance water binding and hydration (Liu et al., 2019). Overall, these results were consistent with SEM observations where pore formation in starch granules under 500 MPa and H conditions facilitated water penetration, thereby contributing to the increased swelling and solubility.

### 3.7 Dynamic shear properties

**Fig. 5:**
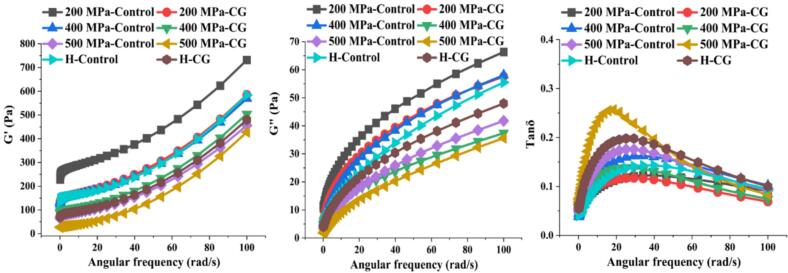
Dynamic shear properties of native rice starch and starch-CG complexes under different treatment conditions.

The rheological properties of rice starch-CG complexes subjected to UHP and H treatments are illustrated in Fig. 5. All samples exhibited typical weak gel behavior, characterized by G′ values greater than G″ across the frequency range of 0 to 100 rad/s, with no crossover observed. Furthermore, the loss tangent (tanδ = G″/G′) values remained consistently below 1. However, under both UHP and H treatments, the addition of CG led to a decrease in G′ and G″ compared with the control. This reduction could be attributed to the release of Ca^2+^ ions from CG, where the negatively charged glucuronic acid groups shield the negative charges on starch molecules, thereby altering the conformation of linear starch chains, reducing molecular entanglement and weakening the starch network. These results aligned with previously reported findings on the rheological properties of CG-induced mung bean starch-linseed protein composite gels (Ji et al., 2021; Min et al., 2023). Additionally, with increasing UHP treatment pressure, both G′ and G″ decreased in the order 200 MPa > 400 MPa > H > 500 MPa. This trend suggested progressive swelling and eventual disintegration of starch granules under pressure, leading to molecular fragmentation as well as reduced viscoelasticity of the starch gels (Chen et al., 2019). The H-treated samples also displayed similar behavior to those treated at 500 MPa, although the 500 MPa treatment caused a more pronounced decrease in G′ and G″. These results suggested that high-temperature treatment could also severely disrupt starch granule structure, compromising gel integrity and strength (Kong et al., 2015).

**Fig. 6:**
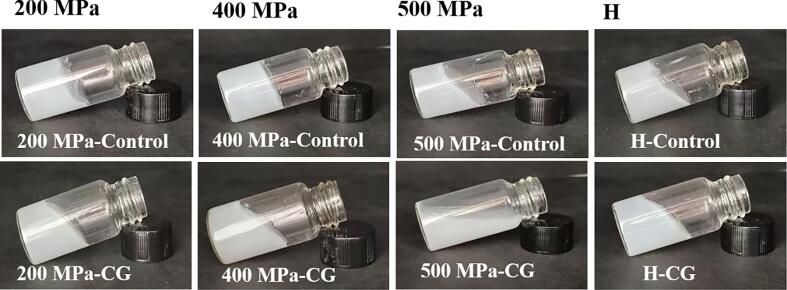
Morphological changes in native rice starch and starch-CG complexes under UHP and H treatments.

The morphology of the starch gels, shown in Fig. 6, suggested that, under UHP treatment, the addition of CG resulted in visibly weakened gel strength. In particular, as the pressure increased, the gels exhibited lower strength but increased fluidity. Similarly, CG addition under H treatment reduced gel integrity. At 500 MPa, the starch gel containing CG exhibited the most severe deformation and highest fluidity, in line with the rheological behavior observed above.

### 3.8 Gel strength

**Fig. 7:**
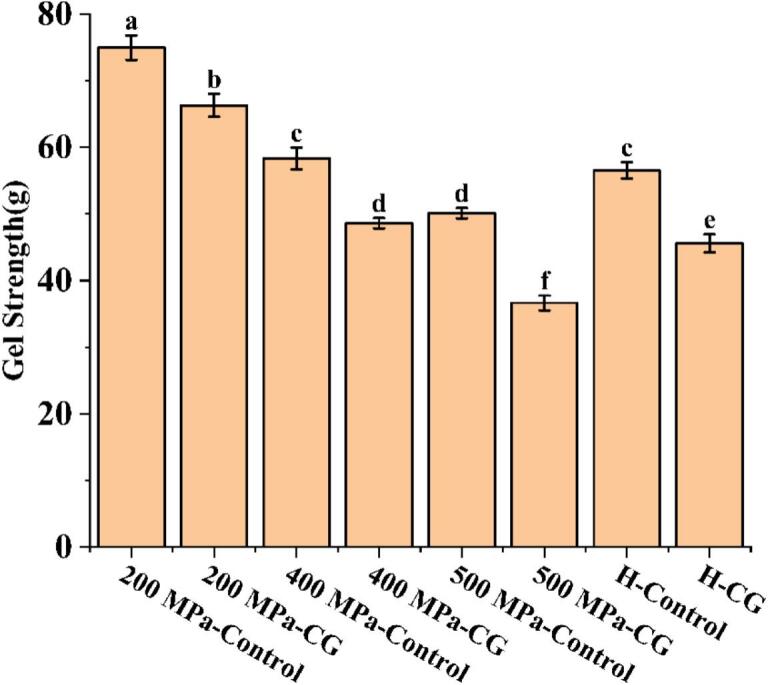
Gel strength of native rice starch and rice starch-CG complexes.

Fig. 7 shows the gel strength of rice starch-CG complexes after UHP and H treatment. Compared to the control group, the addition of CG significantly reduced the gel strength across all treatments. The 500 MPa-CG group showed the lowest value, reaching only 36.65 g. Starch gel strength is primarily governed by the hydrogen bond network formed between molecular chains. The dissociated Ca^2+^ ions from CG act as divalent cations with a stronger affinity for oxygen atoms than for hydrogen atoms. These ions competitively bind to hydroxyl sites on starch molecules that would otherwise participate in hydrogen bonding, consequently weakening both the number and strength of hydrogen bonds between starch chains. This reduction leads to fewer cross-linking points within the gel network, ultimately decreasing gel strength (Fei et al., 2025). The gel strength after the high-pressure treatment (500 MPa) was markedly lower than that after the low-pressure treatments (200 MPa, 400 MPa) or the H treatment. The order of gel strength was as follows: 200 MPa > 400 MPa > H > 500 MPa. This trend can be attributed to the severe disruption of the granular structure and crystalline order of starch under high pressure, which causes starch chain scission, reduces amylose leaching, and hinders the formation of a continuous gel network (Liu et al., 2019; Sherin et al., 2024).

### 3.9 Steady shear viscosity properties

**Fig. 8:**
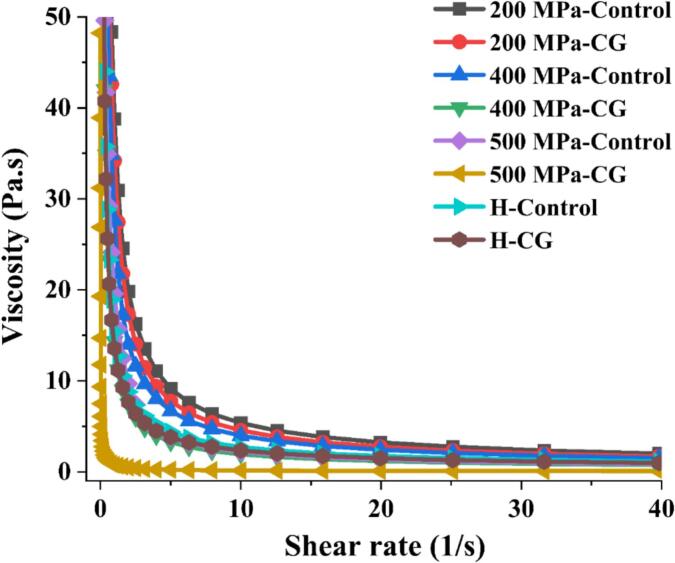
Steady shear viscosity properties of native rice starch and starch-CG complexes.

The steady shear viscosity curves for all samples are presented in Fig. 8. Overall, the samples exhibited shear-thinning behavior, typical of pseudoplastic fluids, with viscosity decreasing as the shear rate increased. After UHP and H treatments, the viscosity of the rice starch-CG complexes was significantly reduced compared with their respective controls in the order 200 MPa > 400 MPa > H > 500 MPa. This reduction in viscosity could be attributed to the release of positively charged Ca^2+^ ions from CG which neutralized the negative charges on starch molecules *via* electrostatic interactions, thereby disrupting the formation of a gel network and limiting amylose leaching (Fei et al., 2025). Furthermore, under identical shear conditions, the shear stress decreased as the treatment pressure increased. Notably, at 500 MPa, the addition of CG markedly lowered the viscosity of starch, suggesting extensive disruption of molecular entanglements within the starch matrix (Chen et al., 2021). The lower viscosity observed in H-treated samples further supported the hypothesis that high temperatures could weaken the interactions between linear starch chains, preventing the formation of a dense, cohesive network structure (Sindhu et al., 2019). These results were consistent with both the rheological properties shown in Fig. 5 and the starch gel morphology in Fig. 6.

### 3.10 Freeze-thaw stability

**Fig. 9:**
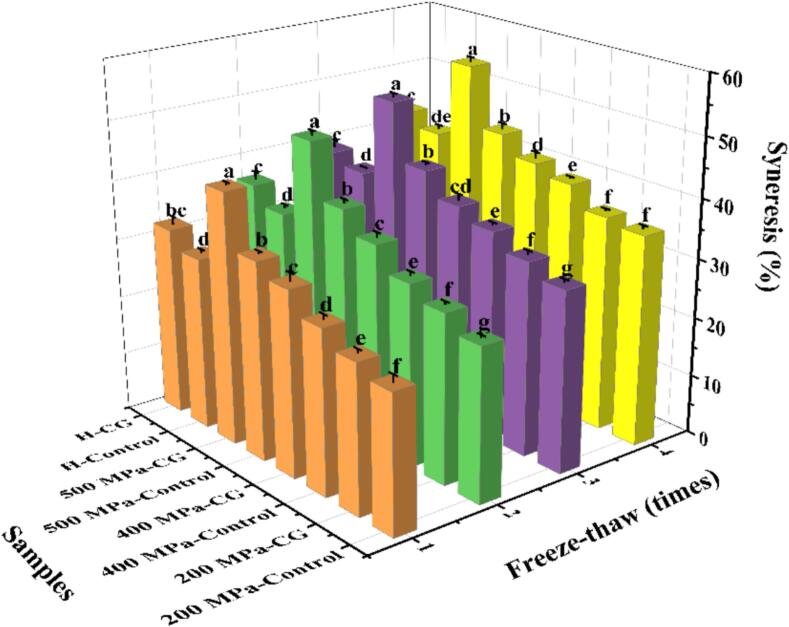
Freeze-thaw stability of native rice starch and starch-CG complexes.

The freeze-thaw stability of all samples is presented in Fig. 9. Repeated freeze-thaw cycles tend to promote the aggregation of linear and branched starch molecules, leading to syneresis. Thus, with an increase in the number of cycles, syneresis progressively increases, indicating a decline in freeze-thaw stability and compromised gel integrity. In this study, starch samples with CG exhibited greater water separation than the controls across all treatment conditions (UHP and H). Notably, the trend in syneresis followed the order: 200 MPa < 400 MPa < H < 500 MPa. This was likely due to CG-induced disruption of the starch gel network, which increased porosity and promoted water migration out of the gel matrix (Abedi & Pourmohammadi, 2020). In addition, syneresis increased with increasing UHP pressure, with the most pronounced water loss of 54.29 % observed in the 500 MPa-CG sample. This could be attributed to the breakdown of both linear and branched starch chains under high pressure, resulting in reduced molecular entanglement that hindered the formation of a stable three-dimensional gel network capable of retaining water during freezing and thawing (Sindhu et al., 2019). Although the H-treated samples displayed similar patterns, water loss was more severe in the 500 MPa group.

### 3.11 *In vitro* digestibility of starch samples

**Fig. 10:**
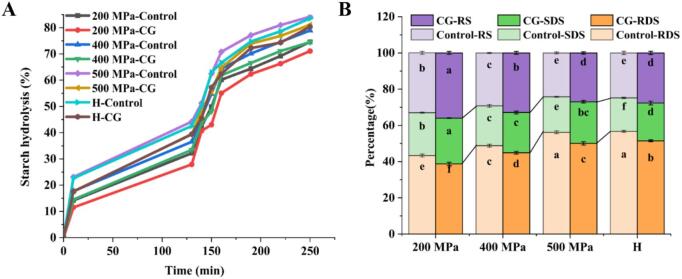
Starch hydrolysis and digestibility characteristics of native rice starch and starch-CG complexes. (A) Hydrolysis profiles (B) RDS, SDS and RS content.

The hydrolysis profiles of rice-starch-CG complexes after UHP and H treatments are shown in Fig. 10(A), with the corresponding levels of RDS, SDS and RS displayed in Fig. 10(B). Table 3 presents the two-factor analysis of treatment conditions (UHP/H treatment) and additive conditions. The results indicate that both processing methods (UHP/H treatment) and CG addition exerted extremely significant effects (*p* < 0.001) on the content of RDS, SDS, and RS in rice starch. Furthermore, a significant interaction between the two factors was observed only for SDS content (*p* = 0.004), while the interaction effects on RDS and RS content did not reach statistical significance. In both UHP and H treatments, the addition of CG decreased the starch hydrolysis rate and RDS content while increasing RS and SDS levels compared with the control. Notably, at 200 MPa, the RS content of the samples reached its peak. The 200 MPa-Control group showed an RS content of 32.98 %, while the 200 MPa-CG group exhibited the highest level at 36.00 %. This may be attributed to a synergistic mechanism between the physical barrier effect of incompletely gelatinized starch granules and the calcium ion-amylose complexation effect. Specifically, starch granules maintained relatively intact structures under 200 MPa pressure, limiting amylase access to the substrates. Concurrently, Ca^2+^ released from the CG may infiltrate starch granules, forming ordered complexes with amylose chains that further enhance resistance to digestion. These results suggested that Ca^2+^ ions released from CG potentially played an inhibitory role in the enzymatic hydrolysis of rice starch and this could have been due to two factors: (1) Ca^2+^ enhanced the RC of starch and promoted the formation of a denser molecular structure that hindered the accessibility of starch to amylase and (2) electrostatic interactions between the surface charge of the amylose‑calcium complex and the active site of amylase site may have altered the enzyme's conformation, reducing its binding efficiency (Yang et al., 2024). Ding, Li, et al. (2023) found that the addition of Ca(OH)₂ increased the RS content in tartary buckwheat starch. Yang et al. (2024) also reported that Ca^2+^ promotes RS formation in black bean starch. These conclusions are consistent with the results of this study. Niu et al. (2019) suggested that changes in starch structure influence RS content. Specifically, calcium ions promote the formation of amylose‑calcium complexes, thereby further increasing RS levels. Additionally, the complexation between starch and calcium ions enhances the structural stability of these complexes. After the addition of calcium ions, the loosely bound amylose released during gelatinization readily undergoes repolymerization with calcium ions, forming a more stable structure that is resistant to enzymatic digestion and thereby effectively reduces starch hydrolysis. Therefore, the addition of CG can effectively suppress glucose release during digestion, which is promising for developing low-glycemic starch-based foods. In addition, as the pressure increased, the hydrolysis rate of starch rose while the RS content declined from 36.00 % (200 MPa-CG) to 24.20 % (500 MPa-Control). Regarding the H treatment group, it showed a similar trend to the 500 MPa treatment one. Overall, these findings demonstrated that both UHP and H-treatments can enhance starch digestibility by disrupting its crystalline structure and breaking the double helix, thereby allowing enzymes to access the starch granules more easily for hydrolyzing the molecules (Oh et al., 2019; Sindhu et al., 2019).

**Table 3:** Two-way ANOVA – *p* values of RDS, SDS, and RS values in rice starch and starch-CG complexes.

Two-way ANOVA – p values	RDS	SDS	RS
Factor A (treatment method (UHP/H))	<0.001	<0.001	<0.001
Factor B (CG addition)	<0.001	<0.001	<0.001
Factor A × factor B	0.128	0.004	0.752

### 3.12 Determination of calcium content

**Fig. 11:**
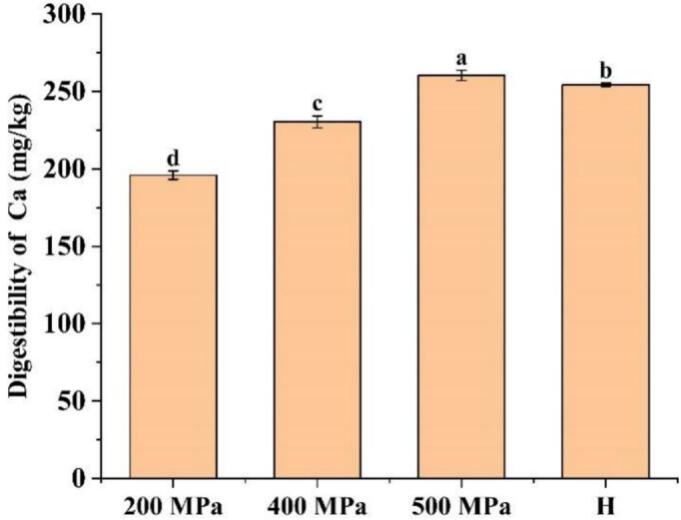
Calcium ion release from starch-CG complexes after *in vitro* digestion.

The release of calcium ions following *in vitro* digestion is shown in Fig. 11. Changes in mineral digestibility can depend on the dissociation of metals previously chelated to macromolecules or their enhanced binding to active sites (Wu et al., 2023). Overall, the calcium ion release increased from 196 mg/kg (200 MPa) to 260.40 mg/kg (500 MPa) under UHP treatment, rising proportionally with pressure. This phenomenon likely results from conformational changes in CG under high pressure (500 MPa), which disrupt its original chelation structure and release bound calcium ions. Additionally, high pressure (500 MPa) induces severe deformation, disintegration, or even gelation of starch granules, thereby compromising their inherent barrier function. As a result, calcium ions embedded within the starch matrix become more exposed to the digestive environment (Wu et al., 2023). However, the H-treated samples exhibited a calcium release of 254.40 mg/kg, slightly lower than that of the 500 MPa group. The increase was likely due to the disruption of the starch crystalline structure under high pressure or heat, with these conditions exposing more binding sites and facilitating the release of calcium ions during digestion (Xia et al., 2017; Zhi et al., 2023). These results were consistent with the trends observed in the *in vitro* digestion of starch-CG complexes.

## 4 Conclusions

In this study, the effects of UHP treatment on rice starch-CG complexes were investigated and compared to those induced by hydrothermal treatment. Both treatments, when combined with calcium ion addition, significantly modified starch properties, leading to reductions in viscosity, gel strength as well as G′ and G″ values, while increasing ΔH*,* RC, solubility, swelling power, gel fluidity, syneresis and RS content. Among these, the 500 MPa treatment resulted in the highest solubility, gel fluidity, swelling power, and syneresis but the lowest viscosity and gel strength, indicating potential for the development of semi-solid foods. Conversely, the 200 MPa-CG group exhibited the highest RS content (36.00 %), suggesting applicability in low-glycemic starch-based foods. Overall, these findings provide valuable insights into the application of UHP treatment for modifying rice starch-CG complexes and highlight its potential for developing semi-solid foods, and food for diabetic individuals. However, further research is required to elucidate the molecular mechanisms underlying the interactions between starch and CG during UHP processing.

## CRediT authorship contribution statement

**Sixuan Zhao:** Writing – original draft, Validation, Software, Formal analysis, Data curation. **Xinhua He:** Writing – review & editing, Methodology, Formal analysis, Data curation. **Yue Wang:** Writing – review & editing, Supervision, Project administration, Methodology, Conceptualization.

## Declaration of competing interest

The authors declare that they have no known competing financial interests or personal relationships that could have appeared to influence the work reported in this paper.

## Data Availability

Data will be made available on request.
